# Spontaneous immune responses against glioma-associated antigens in a long term survivor with malignant glioma

**DOI:** 10.1186/1479-5876-5-68

**Published:** 2007-12-19

**Authors:** Ryo Ueda, Keri L Low, Xinmei Zhu, Mitsugu Fujita, Kotaro Sasaki, Theresa L Whiteside, Lisa H Butterfield, Hideho Okada

**Affiliations:** 1 Department of Neurological Surgery, University of Pittsburgh School of Medicine, 200 Lothrop Street, Pittsburgh, PA 15213, USA; 2 Brain Tumor Program, University of Pittsburgh Cancer Institute, 5117 Centre Avenue, Pittsburgh, PA 15213, USA; 3 Department of Immunology, University of Pittsburgh School of Medicine, 200 Lothrop Street, Pittsburgh, PA 15213, USA; 4 Department of Surgery, University of Pittsburgh School of Medicine, 200 Lothrop Street, Pittsburgh, PA 15213, USA; 5 Department of Dermatology, University of Pittsburgh School of Medicine, 200 Lothrop Street, Pittsburgh, PA 15213, USA; 6 Departments of Pathology and Otolaryngology, University of Pittsburgh School of Medicine, 200 Lothrop Street, Pittsburgh, PA 15213, USA; 7 Department of Medicine, University of Pittsburgh School of Medicine, 200 Lothrop Street, Pittsburgh, PA 15213, USA

## Abstract

**Background:**

In patients with high grade glioma, little is known regarding existence of naturally occurring adaptive T cell reactivity against glioma-associated antigens (GAAs). In this report, we characterized GAA-specific CD8^+ ^T cells and innate immune cells in a patient who has survived with anaplastic astrocytoma (AA) for over 12 years without recurrence.

**Methods:**

Peripheral blood mononuclear cells (PBMCs) derived from the long term survivor with AA were evaluated for the frequency, cytotoxic T lymphocyte (CTL) activity and differentiation status of CD8^+ ^cells recognizing GAA-derived epitopes as well as relative numbers of other immune cell subsets. This patient's AA tissue was evaluated for expression of two GAAs EphA2 and interleukin-13 receptor α2 subunit (IL-13Rα2) by immunohistochemistry.

**Results:**

The patient's tumor expressed both EphA2 and IL-13Rα2, and *in vitro *stimulated PBMC demonstrated superior EphA2_883–891 _and IL-13Rα2_345–353_-specific CTL reactivity compared to PBMC samples from two other patients with progressing malignant glioma. Unstimulated EphA2_883–891_-reactive CD8^+ ^T cells contained high numbers of CD45RA^-^/CCR7^- ^late effector and CD45RA^-^/CCR7^+ ^central memory cells. Among other leukocyte subsets, elevated numbers of NK-T cells were found.

**Conclusion:**

To our knowledge, the current study is one of the first demonstrating the presence of antigen-experienced, GAA-reactive CD8^+ ^T cells in a patient who has survived with AA for over 12 years without recurrence. Further studies are warranted to determine whether the status of GAA-reactive CD8^+ ^T cells dictates survival of patients and/or response to therapeutic vaccines.

## Introduction

Malignant gliomas are the most common type of primary brain tumor, and a major unsolved public health problem with more than 12,000 new cases diagnosed each year in the United States [1]. World health organization (WHO) grade IV glioblastoma multiforme (GBM) is the most common and most malignant of the glial tumors with a median survival of 15 months with the current standard of care, including surgery, radiation therapy and chemotherapy [2]. Anaplastic gliomas (AGs) constitute a group of WHO grade III primary brain tumors including anaplastic astrocytoma (AA), anaplastic oligodendroglioma (AO) [3], anaplastic mixed oligoastrocytoma, and anaplastic ependymoma [4,5]. The prognosis for AGs is still poor. Although AO with chromosome 1p/19q loss respond relatively well to chemotherapy [6,7], median survival for AA patients is approximately 3 years with the multimodal treatment with surgery, radiation and chemotherapy [8].

For cancer-immunosurveillance, cytotoxic T lymphocytes (CTLs) are the key effector cells responsible for immune-mediated destruction of malignant tumors [9,10]. Immunotherapy strategies have been developed to bolster CTL responses against defined tumor-associated antigens in melanoma by antigen-specific vaccination [11,12] and adoptive transfer of CTL [13]. With regard to spontaneous occurrence of CTL responses in cancer patients, long-lasting T cell immune responses against tumor-associated antigens have been well documented in other types of tumors that are considered "immunogenic", such as malignant melanoma [14,15]. However, it remains unclear whether patients with malignant glioma can spontaneously mount antigen-specific T cell responses against glioma-associated antigens (GAAs). This may be at least partially due to the short survival period of these patients.

In addition to CTLs, innate immunity mediated by natural killer (NK) cells and NK-T cells may play significant roles in anti-tumor immunity [16]. Tumor-infiltration of NK cells has been demonstrated to be of positive prognostic values [17]. Further progress of cancer immunotherapy will benefit from better understanding of both innate and adoptive immune response to cancer.

In the current study, we obtained PBMC samples and tumor tissues from a patient who was diagnosed with AA longer than 12 years ago and has had no recurrences since the initial biopsy, radiation therapy and chemotherapy. As median survival for AA patients is approximately 3 years with the current standard therapy [8], these precious samples have provided us with a unique and privileged opportunity to determine whether this patient has mounted spontaneous anti-glioma immune responses. Furthermore, this patient is positive for human leukocyte antigen (HLA)-A2, allowing us to characterize CD8^+ ^T cell immune response against well-characterized HLA-A2-restricted GAA-epitopes, such as EphA2 _883–891 _[18] and IL-13Rα 2 _345–353 _[19], both of which were expressed in this patient's tumor. Our results suggest that this patient may have mounted long-lasting memory response against these GAA-epitopes.

## Methods

### Patients

This study was approved by the local ethical review board of University of Pittsburgh. Three glioma patients signed informed consent before blood samples were obtained. We evaluated PBMC samples derived from the following 3 HLA-A2 patients with high grade glioma (Table 1).

**Table 1:** Clinical histories of glioma patients

Case ID	GBM-1	AA-1	AO-1
Gender	M	F	M
Age at the time of Dx	55	51	58
KPS at the time of Dx	70%	80%	60%
Symptoms	Aphasia	Seizure	Hemi-paresis, seizure
Histology	GBM	AA	AO
Time from Dx to PBMC sampling	24 mos	153 mos	82 mos
Tumor location	Lt. hemisphere	Lt. temporal lobe	Lt. frontal lobe
Treatment	Bx	Bx	Bx, cystic aspiration
	FEBRT	FEBRT	FEBRT
	TMZ/CPT-11 (7 cycles)	BCNU/CDDP/VP-16 (4 cycles)	PCV (3 cycles), TMZ
Time to progression from the Dx	3 mos	No progression for 153 mos	12 mos
Current status of tumor	Progressive disease	No Gd-enhancing lesion	Progressive disease

M; male, F; female, GBM; glioblastoma, AO; anaplastic oligoastrocytoma, mos; months, Bx; biopsy, Dx; diagnosis, KPS; Karnofsky performance status, FEBRT; fractionated external beam radiation therapy, Gd; Gadolinium, TMZ; temozolomide, PCV; procarbazine, lomustine and vincristine, CPT-11; irinotecan, BCNU; bischloroethylnitrosourea, CDDP; cisplatin, VP-16; etoposide.

Patient AA-1 is a 51-year old Caucasian female who presented with sensory seizure in 1994. Biopsy of her left temporal Gadolinium (Gd)-enhancing mass at the Mayo Clinic lead to pathological diagnosis of AA. She was subsequently treated with fractionated external beam radiation therapy (FEBRT) and 4 cycles of chemotherapy consisting of BUNU (bischloroethylnitrosourea), cisplatin and etoposide. She demonstrated a complete disappearance of the Gd-enhancing lesion on MRI, and to date, has had no evidence of tumor recurrence based regular follow-up MRI scans.

Patient GBM-1 is a 55-year old Caucasian male who presented with motor aphasia in 2005. MRI demonstrated mulitfocally enhancing masses in his left hemisphere, and biopsy in University of Pittsburgh revealed GBM. The patient subsequently received FEBRT and concurrent chemotherapy with temozolomide (TMZ), followed by adjuvant chemotherapy using TMZ and CPT-11 for 7 cycles before progression was confirmed by MRI at 6 months after the biopsy.

The other patient AO-1 is a 58-year old Caucasian male who presented with right hemi-paresis in 2000. Biopsy of ring-enhanced cystic lesion in his left frontal lobe led to pathological diagnosis of AO. Despite FEBRT and subsequent 3 cycles of chemotherapy with PCV, progression was found at 1 year after the diagnosis. Although the patient demonstrated partial response to the 2^nd ^line chemotherapy with TMZ, the Gd-enhancing lesion remained as stable disease with continued TMZ therapy when blood samples were drawn.

### Cell lines

The SNB19 glioma cells were cultured in DMEM supplemented with 10% fetal bovine serum (FBS), 100 IU/mL penicillin, 100 μg/mL streptomycin, and 10 mmol/L L-glutamine (all reagents from Invitrogen, Carlsbad, CA) [19]. The transporter associated with antigen processing-deficient, HLA-2.1^+ ^T2 cell line was maintained in RPMI 1640 (Invitrogen) supplemented with 10% FBS and 100 IU/mL penicillin, 100 μg/mL streptomycin, and 10 mmol/L L-glutamine (all reagents from Invitrogen) [19].

### Immunohistochemical staining

Paraffin-embedded tissue sections (10 μm) were deparaffinized in xylene and rehydrated in a series of ethanol/PBS washes. Antigenicity was retrieved with modified citrate solution (Dako) in a pressure cooker for 20 minutes. Endogenous peroxidase was blocked by incubation in 3.0% hydrogen peroxide in PBS, and the nonspecific binding of antibodies was blocked by incubation in serum-free blocking solution (Dako) for 1 hour. The slides were then incubated with anti-human EphA2 monoclonal antibody (1:100; Ab 208, mIgG1, MedImmune) or anti-human IL-13Rα2 polyclonal antibody (15 μg/ml; AF146, gIgG, R&D) diluted in the same blocking solution overnight at 4°C. The slides were incubated with goat anti-mouse (1:200; Upstate Cell Signaling) or rabbit anti-goat (1:200; Abcam) secondary antibodies for 1 hour at room temperature. HRP labeling was visualized using Vectastain's Nova Red kit. The sections were lightly counterstained with Gill's hematoxylin.

### Peptides

The synthetic peptides IL-13Rα2 (345–353, WLPFGFILI; 345-1A9V, ALPFGFILV), EphA2 (883–891, TLADFDPRV) and Influenza M1 (58–66, GILGFVFTL) were synthesized by *N*-(9-fluorenyl) methoxycarbonyl chemistry in the University of Pittsburgh Peptide Synthesis Facility and were >95% pure as indicated by analytic high-performance liquid chromatography and mass spectrometric analysis. Peptides were dissolved in PBS/10% DMSO at a concentration of 2 mg/mL and stored at -20°C until use.

### HLA-A2/peptide tetramer staining

Phycoerythrin (PE)-conjugated human leukocyte antigen (HLA)-A*0201/ALPFGFILV tetramer (IL-13R2-tetramer) and PE-conjugated HLA-A*0201/TLADFDPRV tetramer (EphA2-tetramer) were produced by the National Institute of Allergy and Infectious Disease tetramer facility within the Emory University Vaccine Center (Atlanta, GA) using the peptide synthesized by the University of Pittsburgh Peptide Production Facility. Cells were stained with tetramers (10 μg/mL) in PBS containing 1% bovine serum albumin for 15 minutes at room temperature, washed once, and stained with fluorescein isothiocyanate (FITC)-conjugated anti-human CD8 (BD Biosciences, San Diego, CA). Flow cytometric analyses were done in the University of Pittsburgh Cancer Institute (UPCI) Flow Cytometry Facility on a DakoCytomation CyAN 9-Color High Speed Analyzer using Dako Summit analysis program.

### Four-color flow cytometry phenotype Analysis

PBMCs from the glioma patients were stained with several different combinations of monoclonal antibodies that have been used to delineate subsets of human memory/effector CD8^+ ^T cells. Each sample (2 × 10^6 ^PBMCs) was stained with PE-conjugated EphA2-tetramer, FITC-conjugated anti-human CD8 (BD Biosciences), phycoerythrin-cyanine 7-conjugated anti-human chemokine receptor (CCR) 7 (BD Biosciences), and ECD-conjugated anti-human CD45RA (BD Biosciences). After the addition of the monoclonal antibodies, cells were incubated at 4°C for 30 min, washed twice in staining buffer, and fixed in 500 μl of 1% paraformaldehyde. Cells were stored in the dark at 4°C until analysis, which occurred within 24 h of staining. All analyses were performed on four-color FACS using Summit software (Dako Colorado, Inc. Fort Collins, CO). Appropriate compensation and isotype controls were run for all samples.

### *In vitro *induction of CTL in patient-derived PBMCs

To generate dendritic cells (DCs), the plastic adherent PBMCs were cultured in AIM-V medium (Invitrogen) supplemented with 1,000 units/mL recombinant human granulocyte macrophage colony-stimulating factor and 500 units/mL recombinant human IL-4 (rhIL-4; Cell Sciences) at 37°C in a humidified CO_2 _(5%) incubator. Six days later, the immature DCs were stimulated with recombinant human tumor necrosis factor-α, IL-6, and IL-1β (10 ng/mL each). Mature DCs were then harvested on day 8, resuspended in AIM-V medium at 1 × 10^6 ^per mL with peptide (10 μg/mL), and incubated for 2 hours at 37°C. Populations of autologous CD8^+ ^T cells were enriched from PBMCs using magnetic microbeads (Miltenyi Biotech, Auburn, CA). CD8^+ ^T cells (2 × 10^6 ^per well) were cocultured with 2 × 10^5 ^per well peptide-pulsed DCs in 2 mL/well of AIM-V medium supplemented with 5% human AB serum, 10 units/mL rhIL-2 (R&D Systems, Minneapolis, MN), and 10 units/mL rhIL-7 (Cell Sciences) in each well of 24-well tissue culture plates, Multiwell Primaria (Falcon). On day 15, lymphocytes were restimulated with autologous DCs pulsed with peptide in AIM-V medium supplemented with 5% human AB serum, rhIL-2, and rhIL-7 (10 units/mL each). On day 20, the CD8^+ ^cultured cells were analyzed for CTL activity by standard 4-hour ^51^Cr release assay.

### ^51^Cr release cytotoxicity assay

Target cells (1 × 10^4 ^cells in 100 μl) labeled with 50 μCi of Na^51^CrO_4 _(^51^Cr) were added to wells containing varying numbers of effector cells (100 μl) using U-bottomed 96-well plates. After 4 h incubation at 37°C, cells were centrifuged and 30 μl supernatant was collected and measured for radioactivity. Percentage of specific lysis (% specific lysis) was calculated as using triplicate samples follows: percentage lysis = (cpm experimental release-cpm spontaneous release)/(cpm maximal release-cpm spontaneous release) × 100.

### Evaluation of leukocyte subsets in PBMCs

To obtain absolute lymphocyte counts (cells/μL) for each desired lymphocyte subset, fresh peripheral blood samples were subjected to the Q-Prep/Prep Plus 2 system (Beckman-Coulter). Monoclonal antibodies, CD3-FITC, CD4-PC5, CD8-ECD, CD25-PE, and CD19-ECD, and isotype controls, IgG_1_-FITC, IgG_2a_-PE, and IgG_1_-ECD were purchased from Beckman-Coulter. CD16-PC7 and CD56-PC7, and isotype controls, IgG_1_-PC7 IgG_2b_-PC7 were purchased from Becton-Dickinson.

After the cells were stained with these monoclonal antibodies, "Flow Count" fluorospheres (Beckman-Coulter) were added to each specimen using the Q-Prep/Prep Plus 2 for dispensing. These beads have a uniform size, fluorescence and concentration, allowing for direct measurement of absolute counts. The lymphocytes were gated using forward vs. side scatter, and each subset with specific phenotype was evaluated for both percentages of lymphocytes analyzed and the absolute count. Expected normal control values were based on data with normal individuals.

### Statistical analysis

Data are presented as means and standard deviation. Differentiation status of EphA2-tetramer^+^/CD8^+ ^T cells were analyzed by one way analysis of variance for comparing means of three or more variables, ANOVA.

## Results

### Expression of GAAs in the tumor derived from AA-1

**Figure 1:**
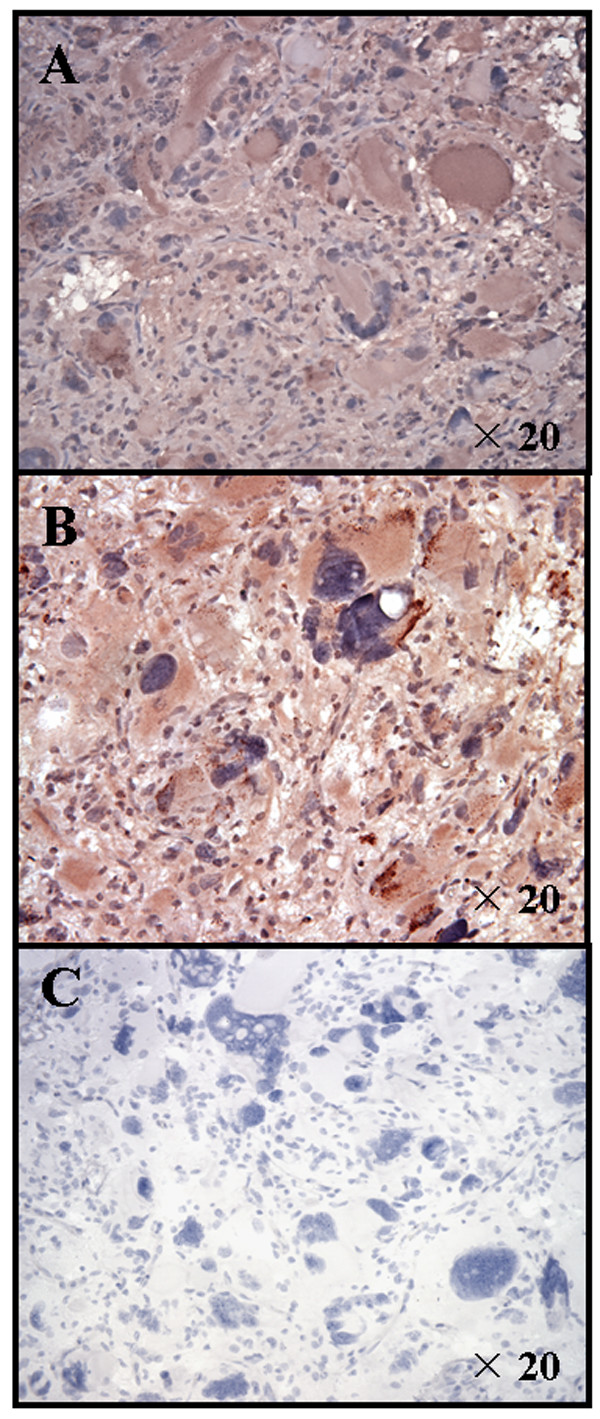
**Anaplastic astrocytoma of the glioma long term survivor (AA-1) expressed IL-13Rα 2 and EphA2**. Paraffin-embedded sections were stained with anti-human IL-13Rα 2 polyclonal antibody (***A***), anti-EphA2 mAb (Ab208 mIgG1) (***B***), or without primary antibody (***C***) as described in Materials and Methods. Original magnification was × 20.

We evaluated expression of IL-13Rα2 and EphA2 in tumor tissues derived from the patient AA-1, as these GAAs encode well characterized HLA-A2-restricted CTL epitopes [18,19]. Immunohistochemical analyses of paraffin-embedded specimens demonstrated a diffuse membranous, partially cytoplasmic staining pattern in glioma cells for both IL-13Rα2 and EphA2 (Figure 1A and 1B, respectively). In contrast, negative control sections stained without primary antibodies exhibited a negative or minimal staining (Figure 1C).

### *In vitro *stimulation of AA-1-derived PBMC induces potent GAA-specific CTLs

**Figure 2:**
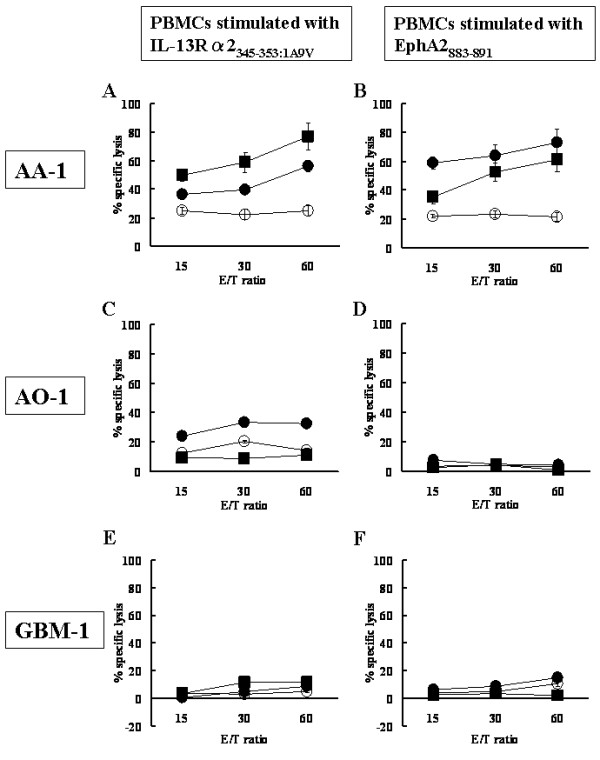
***In vitro *stimulation of AA-1-derived PBMC efficiently induced GAA-specific CTLs**. PBMCs derived from HLA-A2^+ ^glioma patients, AA-1, AO-1, and GBM-1, were stimulated with autologous DCs loaded with IL-13Rα 2_345–353:1A9V _(***A, C and E***) or EphA2_883–891_(***B, D and F***). On day 20 after the primary stimulation, responder cells of AA-1 (***A, B***), AO-1 (***C, D***), and GBM-1 (***E, F***) were tested for their lytic ability against human glioma cells SNB19 (Solid square, HLA-A2^+^, EphA2^+^, IL-13Rα 2^+^), or T2 cells loaded with IL-13Rα 2_1A9V _(Solid circle in ***A, C and E***), EphA2_883–891 _(Solid circle in ***B, D and F***) or T2 cells loaded with Influenza M1_58–66 _(hollow circle) using 4-hour ^51^Cr-release assays. Values indicate averages of duplicated samples, and represent data from one of two experiments with similar results. Bars indicate standard errors.

Expression of IL-13Rα 2 and EphA2 in the patient AA-1's tumor led us to address whether antigen-specific T cell response had been mounted in this patient. We first determined whether AA-1-derived PBMC could demonstrate efficient antigen-specific CTL reactivity against IL-13Rα2_345–353 _and EphA2_883–891 _following short-term *in vitro *re-stimulation. PBMCs derived from three donors, AA-1, GBM-1 and AO-1, were stimulated with autologous DCs loaded with EphA2_883–891 _or IL-13Rα2_345–353:1A9V _and evaluated for antigen-specific cytotoxic activity by ^51^Cr release assays (Figure 2). AA-1 derived effector cells efficiently lysed T2 cells pulsed with the relevant peptide, but not control T2 cells pulsed with irrelevant M1_58–66 _peptide, demonstrating antigen-specific CTL activity of the effector cells. Furthermore, these effector cells efficiently lysed SNB19 human glioma cells that endogenously express HLA-A2, EphA2 and IL-13Rα 2 [18,20]. In contrast, effector cells obtained from AO-1 and GBM-1 were unable to lyse any of the target cells tested, except that effector cells from AO-1 demonstrated a mild CTL activity against IL-13Rα2_345–353_-pulsed T2 cells. These data suggest that the patient AA-1 has mounted and maintained CTL precursors that can exert antigen-specific CTL reactivity upon re-stimulation.

### Antigen-experienced, memory phenotype of EphA2-reactive CD8^+ ^cells derived from the glioma long term survivor

**Figure 3:**
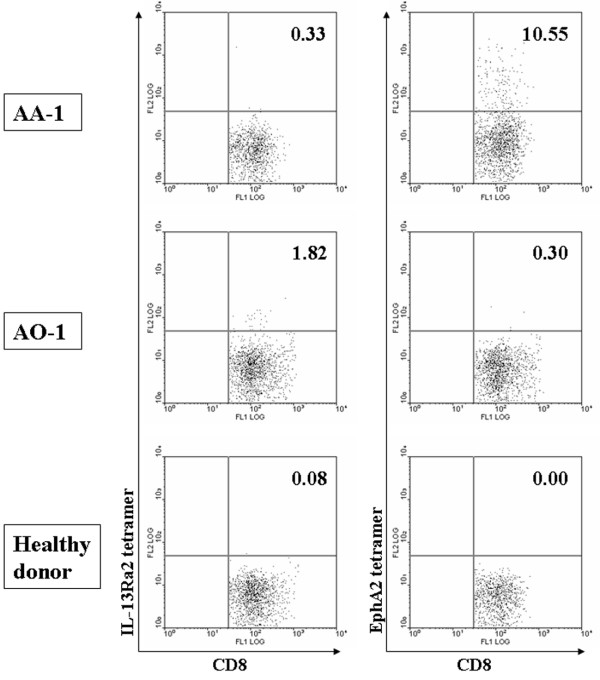
**AA-1 derived PBMC contained high numbers of EphA2_883–891_-tetramer^+^/CD8^+^cells**. Cryopreserved PBMCs derived from AA-1 (upper panels), AO-1 (middle panels), and an HLA-A2^+ ^healthy donor (lower panels) were double-stained with FITC-conjugated anti-CD8 mAb and PE-conjugated IL-13Rα 2_1A9V_-tetramer (left columns) or EphA2_883–891_-tetramer (right columns). Cells sorted for CD8^+ ^by flow cytometry are displayed. Numbers in each histogram indicate the percentage of tetramer-reactive cells among CD8^+ ^cells. In three independent assays with AA-1-derived PBMC samples obtained at three separate time points spanning six months, the percentage of EphA2_883–891_-tetramer-reactive CD8^+ ^cells was found to be 5% to 10.5% in CD8^+ ^PBMC.

Efficient induction of the CTL response by *in vitro *re-stimulation could reflect high numbers of naïve and/or memory GAA-specific CD8^+ ^cells present in the patient AA-1's PBMC. We therefore conducted flow cytometric analyses of GAA-specific CD8^+ ^cells in non-stimulated PBMC derived from AA-1. Although we detected only background levels of CD8^+ ^cells reactive to HLA-A*0201-IL-13Rα2_345–353:1A9V_-tetramer [19], higher frequencies of HLA-A*0201-EphA2_883–891_-tetramer^+^/CD8^+ ^cells were detected in PBMCs derived from the patient AA-1 compared to those from the patient AO-1 and a healthy donor (Figure 3). Although patient AO-1 exhibited the highest frequency of IL-13Rα2-tetramer^+^/CD8^+ ^cells compared to two other donors, the CTL response following *in vitro *stimulation was only modest against this epitope as shown in Figure 2, suggesting a functional deficiency of IL-13Rα2-reactive CD8^+ ^cells in this patient.

**Figure 4:**
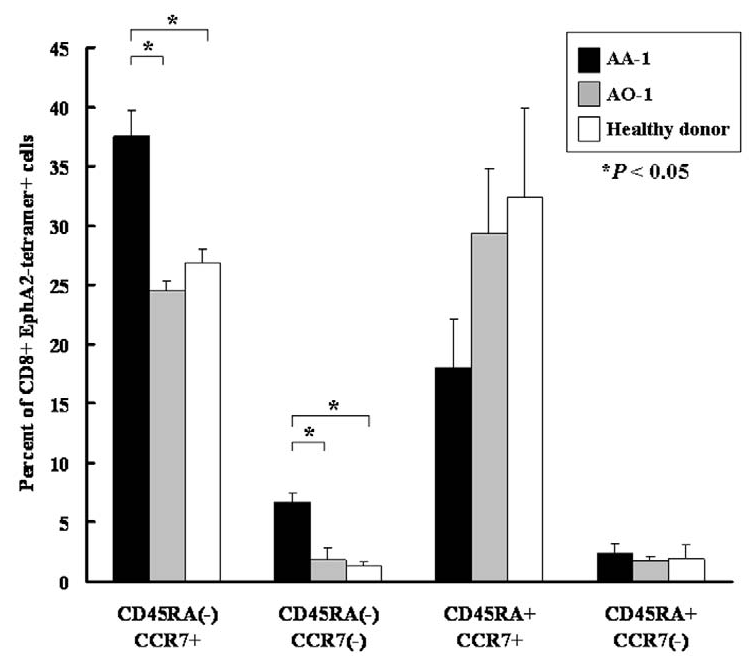
**A majority of EphA2_883–891_-reactive CD8^+ ^cells in AA-1-derived PBMC are central memory cells**. Freshly thawed PBMCs were stained with anti-CD8 mAb, EphA2_883–891_-tetramer, anti-CD45RA mAb, and anti-CCR7 mAb and analyzed as described in Materials and Methods. *Black, gray *and *white columns *indicate the percentage of each population in EphA2_883–891_-specific CD8^+ ^cells derived from AA-1, AO-1, and healthy donor, respectively. Bars indicate standard errors. Higher percentages of central memory T cells (CD45RA^-^/CCR7^+^) and effector memory T cells (CD45RA^-^/CCR7^-^) were observed in EphA2-tetramer^+^/CD8^+ ^T cells of AA-1 compared to those of AO-1 and the healthy donor (*P *< 0.05; ANOVA).

We further examined differentiation phenotype of the EphA2-tetramer^+^/CD8^+ ^T cells based on the status of CC chemokine receptor 7 (CCR7) and CD45RA [21]. Higher percentages of central memory T cells (CD45RA^-^/CCR7^+^) and effector memory T cells (CD45RA^-^/CCR7^-^) were observed in EphA2-tetramer^+^/CD8^+ ^T cells of AA-1 compared to those of AO-1 and the healthy donor (*P *< 0.05; ANOVA, Figure 4). On the other hand, there was a trend towards lower numbers of naïve T cells (CD45RA^+^/CCR7^+^) among EphA2-recognizing CD8^+ ^cells in AA-1 compared to AO-1 and the healthy donor, although this difference did not reach a statistical significance. These results suggest that the long term survivor AA-1 may have mounted an EphA2-specific CD8^+ ^T cell response, and that a majority of these cells had differentiated *in vivo *towards antigen-experienced, central memory or effector memory cells.

### Elevated NK-T cell numbers in PBMC obtained from the patient AA-1

To characterize the frequencies of other immune cells in the patient AA-1, we evaluated leukocyte subsets in whole blood using T cell markers CD3, CD4, and CD8, a B-cell marker (CD19), NK cell markers (CD16 and CD56) as well as an activation marker CD25. Compared to the normal range obtained from normal donors, PBMC of AA-1 demonstrated elevated NK-T (CD3^+^/CD16^+^/CD56^+^) and NK (CD3^-^/CD16^+^/CD56^+^) cell populations (Table 2), suggesting a possibility that these innate immune effector cells may have contributed to the long term anti-tumor immune surveillance in this host. To eliminate concerns over natural variation due to environment, this analysis was repeated three times (at 2, 4 and 6 months following the initial assay) with identical results.

**Table 2:** Lymphocyte populations in PBMC derived from AA-1

	Counts (Number/μl)	
	<AA-1>	<Normal range>
CD3^+ ^(T cell)	1130.3 ± 193.4	912–1973
CD3^+^/CD4^+ ^(CD4^+ ^T cell)	799.7 ± 134.2	527–1376
CD3^+^/CD8+ (CD8^+ ^T cell)	363.7 ± 65.6	290–604
CD3^+^/CD16^+^56^+ ^(NK T cell)	175.7 ± 24.7	17–131
CD3^-^/CD16^+^56^+ ^(NK cell)	276.3 ± 20.2	59–252
CD19^+ ^(B cell)	242.3 ± 38.3	104–383
CD4^+^/CD25^+ ^(Activated T cell)	182.3 ± 19.2	131 – 539
CD4^+^/CD25^high+^	58.7 ± 18.7	31 – 115

## Discussion

It has been well documented in immunogenic cancers, such as melanoma [22-24], that cancer-bearing hosts can spontaneously mount CTL responses against tumor associated antigens detectable in peripheral blood and metastatic nodes. It has also been suggested that naturally occurring CTL responses may be associated with protection from tumor recurrence in melanoma long term survivors [15,25]. However, in patients with malignant glioma, to our knowledge, the current report is one of the first evaluating a naturally occurring immune response, particularly an adaptive CD8^+ ^T cell response against two well-characterized GAA-epitopes. Wheeler *et al. *have demonstrated that levels of CD8^+ ^recent thymic emigrants (RTEs), typically a tiny proportion of CD8^+ ^T cells, constitute the majority of tumor antigen-binding small precursor cells in PBMC from GBM patients and account for the prognostic power of age on clinical outcome in GBM patients [26]. Even though our current study points to the significance of antigen-experienced GAA-reactive cells, it will be intriguing to evaluate RTEs in PBMC from the patient AA-1.

The frequency of EphA2_883–891_-reactive CD8^+ ^T cells in the patient AA-1 was remarkably high (Figure 3). In other cancers, including melanoma [27], hepatocellular carcinoma [28,29] and breast cancer [30], increased frequencies of tetramer-reactive CD8+ cells have been reported, although none of these studies found as high frequencies of tetramer-reactive cells as found in our current study. What is quite intriguing in these previous studies is that they all suggest or indicate the presence of nonfunctional tetramer^+^/CD8^+ ^T cells in circulating PBMC. In our case, comparable levels of antigen-specific CTL were induced against both IL-13Rα 2 and EphA2 (Figure 2) despite there were remarkably higher numbers of EphA2-tetramer^+^/CD8^+ ^cells than IL-13Rα 2-tetramer^+^/CD8^+ ^cells, suggesting a possibility that EphA2-tetramer^+^/CD8^+ ^cells have contained some nonfunctional cells.

Further characterization of EphA2_883–891_-reactive CD8^+ ^T cells revealed a majority of these cells are antigen-experienced, which were skewed towards central memory or effector memory cells (Figure 4). Given the fact that the patient AA-1's tumor tissue expressed high levels of EphA2, it is conceivable that tumor-expression of EphA2 has triggered activation of EphA2-reactive CD8^+ ^precursor cells, of which the majority remained as effector memory and central memory T cells. It is noteworthy that central memory tumor-reactive CD8^+ ^T cells confer efficient and persistent antitumor immunity in preclinical tumor models [31,32]. It remains controversial, however, as to whether maintenance of tumor-specific memory T cells requires the presence of tumor-antigens in the host [33]. In the current study, patient AA-1 has not had a tumor-recurrence for over 12 years. This does not exclude, however, a possibility that residual tumor cells still persist in the host, and have provided the host immunity with a source of antigen. Indeed, all malignant gliomas grow invasively to the surrounding brain, and it is not considered possible to eliminate all tumor cells even if MRI scans do not detect Gd-enhancing lesion in the brain of patients. Nonetheless, our results suggest a possibility that the high frequency of GAA-specific central memory T cells may have contributed to the long term protection of the patient AA-1 from tumor recurrence.

It would also be intriguing to delineate molecular mechanisms underlying the antigen presentation against EphA2- and IL-13Rα 2-derived epitopes in the patient AA-1 that led to anti-tumor immunity instead of tolerance. Recently, Apetoh *et al. *has shown that patients with Toll-like receptor (TLR)-4 mutation do not mount a chemotherapy-primed anti-tumor immune response, whereas intact TLR-4 binds high mobility group box 1 (HMGB1), an early mediator of inflammation, to activate DCs [34]. It would be interesting to determine the status of TLR-4 in AA-1, versus the other patients in this study.

The elevated NK-T (CD3^+ ^CD16^+ ^CD56^+^) and NK (CD3^- ^CD16^+ ^CD56^+^) cell populations in the patient AA-1 (Table 2) suggest that these innate effector cells may also have participated in the protection of tumor-recurrence in this patient. However, we were unable to demonstrate a significantly superior cytotoxic activity of NK cells isolated from the patient AA-1 compared to those isolated from two other donors, GBM-1 and AO-1 (data not shown). Further studies are warranted to evaluate the functional status of NK-T cells in the patient AA-1 as NK-T cells can compose an important part of anti-tumor host immunity [16].

In the current study, as controls for the samples from AA-1, we tested PBMC from two other patients with malignant glioma, both of which had recurrence of their tumors and had received second-line chemotherapy by the time their blood samples were drawn. One may raise a concern as to whether our observations with the patient AA-1 are significant because direct comparisons were made only with these two other donors. In our previous studies with PBMC samples from a total 42 patients with malignant glioma [19], stimulation with the IL-13Rα 2 _345–353:1a9V _peptide induced antigen-specific CTL responses in 18 of 42 (42.9%) cases. Interestingly, we could not detect any statistically significant correlations between positive responsiveness and clinical variables, such as patient age (>50 or <50), type of glioma (glioblastoma multiforme versus others), and prior or current treatment (i.e., chemotherapy). As we used identical stimulation conditions in the current study, poor CTL responses observed in the two direct control donors (GBM-1 and AO-1) may not represent results that would be expected in patients with malignant glioma in general. Nevertheless, in these two patients with histories of recurrences, low levels of EphA2-reactive CD8^+ ^T cells were associated with weak induction of GAA-specific CTL responses, suggesting a possibility that tetramer-based assessment of GAA-specific CTL precursors may dictate the host-response to subsequent stimulations with GAA, such as GAA-based vaccines.

## Conclusion

Our data generated with blood and tumor samples from patient AA-1 are highly valuable as it is uncommon to find long term survivors with malignant glioma, especially without recurrences for over 10 years [8]. The HLA-A2 haplotype allowed us to study GAA epitope-specific T cell responses in this patient. Potent induction of CTL and presence EphA2-specific memory CD8^+ ^T cells observed in the current study suggest that GAA-specific immunity may serve as one critical surveillance mechanism against recurrence and progression of malignant glioma.

## Authors' contributions

RU carried out CTL assays and flow-cytometric analyses. RU played a central role in the preparation of this manuscript. KLL prepared reagents and performed immunohistochemical analyses of tumor sections. XZ, MF and KS provided technical expertise in flow-cytometric evaluation of PBMCs. TLW and LHB supervised and conducted analyses of lymphocyte-subsets in PBMCs. HO conceived of the study, and participated in its design and coordination. All authors have read and approved the final manuscript.
